# Exploring the effects of real-time online cardiac telerehabilitation using wearable devices compared to gym-based cardiac exercise in people with a recent myocardial infarction: a randomised controlled trial

**DOI:** 10.3389/fcvm.2024.1410616

**Published:** 2024-06-05

**Authors:** A. Mitropoulos, M. Anifanti, G. Koukouvou, A. Ntovoli, K. Alexandris, E. Kouidi

**Affiliations:** ^1^ Laboratory of Sport Medicine, Department of Physical Education and Sport Science, Aristotle University of Thessaloniki, Thessaloniki, Greece; ^2^ Lifestyle, Exercise and Nutritional Improvement (LENI) Research Group, Department of Nursing and Midwifery, Sheffield Hallam University, Sheffield, United Kingdom; ^3^ Laboratory of Management of Sports Recreation and Tourism, Department of Physical Education and Sport Science, Aristotle University of Thessaloniki, Thessaloniki, Greece; ^4^ Department of Physical Education, Sports Sciences Frederick University, Nicosia, Cyprus

**Keywords:** online exercise, home-based exercise, coronary heart disease, myocardial infarction, cardiac rehabilitation

## Abstract

**Background:**

Exercise-based cardiac rehabilitation (CR) is a non-pharmacological multidisciplinary programme for individuals after myocardial infarction (MI) that offers multiple health benefits. One of the greatest barriers to CR participation is the travel distance to the rehabilitation centre. Remotely monitored CR appears to be at least as effective in improving cardiovascular risk factors and exercise capacity as traditional centre-based CR. Nevertheless, the efficacy of remotely monitored CR in individuals with a recent MI has yet to be examined.

**Methods:**

A total of 30 individuals (8 women, 22 men) after a recent (i.e., <4 weeks) MI were randomly allocated into two groups (online home-based and gym-based groups). Both groups underwent a 26-week CR programme three times per week. All patients performed baseline and 24-week follow-up measurements where peak oxygen uptake (VO_2peak_), mean daily steps, distance, and calories were assessed.

**Results:**

The online group showed an improvement in mean daily steps (*p* < 0.05) and mean daily distance (*p* < 0.05) at 24 weeks compared to the gym-based group. The paired-sample *t*-test showed that all the assessed variables were statistically (*p* < 0.001) improved for both groups at 24 weeks. Pearson's *r* demonstrated positive correlations between VO_2peak_ and mean daily distance (*r* = 0.375), and negative correlations between VO_2peak_ and muscle (*r* = −0.523) and fat masses (*r* = −0.460). There were no exercise-induced adverse events during the study.

**Conclusion:**

Our findings might indicate that a real-time online supervised CR exercise programme using wearable technology to monitor the haemodynamic responses in post-MI patients is equally effective as a gym-based exercise programme.

## Introduction

Myocardial infarction (MI) is a life-threatening coronary event (1) and the most severe clinical manifestation of coronary artery disease (CAD) (2). In 2020, cardiovascular disease (CVD) globally accounted for 19.05 million deaths, which corresponded to an increase of 18.71% from 2010 (3). The 2020 Hellenic Statistical data revealed that nearly 35% of all deaths in Greece were attributed to CVD, such as stroke and ischaemic heart diseases (4). In the Greek population, 8 out of 10 adults have at least one major modifiable risk factor (e.g., diabetes, hypercholesterolemia, hypertension, smoking, and physical inactivity) for CVD (5). The overall age-adjusted prevalence of MI in the Greek population was found to be 3.6% (5). It was estimated that in adults, approximately 8.7% of all-cause mortality was attributed to a physical activity (PA) level lower than the recommended levels (3).

Exercise-based cardiac rehabilitation (CR) is a non-pharmacological, multidisciplinary, programme for individuals after MI that offers multiple health benefits. For example, CR is able to improve cardiorespiratory fitness (CRF), quality of life (QoL), and exercise capacity, as well as reduce morbidity and mortality (6). More specifically, a large research clinical trial (*n* = 359 people with acute MI) demonstrated that a 6-week hospital- or home-based CR programme significantly improved the resting heart rate (HR), peak oxygen uptake (VO_2peak_), total exercise duration, and metabolic equivalents regardless of obesity (7).

The global consensus recommendations for CR programmes after MI indicate that these programmes should be offered to all affected patients (8). Despite the proven efficacy of the CR programmes and the international consensus for their implementation, participation, and adherence rates to these programmes are low. A recently published study in Greece demonstrated that the greatest barriers seem to be distance from the rehabilitation centre, costs, lack of information about CR, and the fact that some patients perform exercise at home (9). To overcome the barriers of distance and related travel costs, a well-structured, safe, and feasible cardiac telerehabilitation programme is warranted.

Recent evidence indicates the potential for remotely monitored CR for people with cardiac conditions (10). That is, remotely monitored CR appears to be at least as effective in improving cardiovascular risk factors and exercise capacity as traditional centre-based cardiac rehabilitation (11). Several studies have utilised wearable sensors [e.g., HR and electrocardiogram (ECG) monitors] to remotely monitor the intensity and safety of the exercise protocol in people with cardiac conditions (10). Nevertheless, the efficacy of a remotely monitored CR in people with recent MI has yet to be examined.

The aim of this randomised controlled trial was to compare a real-time home-based cardiac telerehabilitation programme monitored by wearable devices in people with recent MI against a traditional gym-based cardiac rehabilitation programme. To our knowledge, this is the first clinical trial to perform CR utilising both telemonitoring and real-time online tele-coaching in people with recent MI. It was hypothesised that the combination of telemonitoring (i.e., wearable devices) and tele-coaching (i.e., a real-time online CR instructor) will allow us to safely balance the intensities of home- and gym-based CR exercise programmes.

## Methods

### Study design

This was a single-centre, pragmatic, double-blinded (e.g., assessor pre- and post-measurements, independent statistician), two-arm randomised controlled trial conducted in Thessaloniki, Greece. We recruited 30 people (8 women, 22 men) after a recent (i.e., <4 weeks) MI with a stable clinical status. The exclusion criteria consisted of the following: (1) unstable angina; (2) acute heart failure (HF); (3) severe heart failure with left ventricle ejection fraction (LVEF) <30%; (4) malignant ventricular arrhythmias; (5) uncontrolled arterial hypertension; (6) musculoskeletal or neurological impairments; and (7) psychological or cognitive disorders that may affect their participation in the exercise programmes. Eligible participants were recruited from the Cardiology Clinics of the University and private Hospitals of Thessaloniki, Greece, and from private physicians’ practices. All patients provided written consent to participate. The Research Ethics Committee of the School of Physical Education and Sport Science of Thessaloniki (Greece) approved the study protocol, and the study complied with the Declaration of Helsinki. The study has also been registered on ClinicalTrial.gov (ID: NCT06071273).

### Patient and public involvement

Although the current study explores the effectiveness of an online CR programme for post-MI patients, we did involve post-MI patients in the design of our methods and their preferences for exercise modalities. That is, our baseline and follow-up assessment protocol (i.e., peak oxygen uptake test) has been shaped to include the minimum core assessments that will provide a response concerning the effectiveness of our protocol and that will not increase the clinical burden (i.e., prolonged assessments) for our participants based on their preferences.

**Figure 1:**
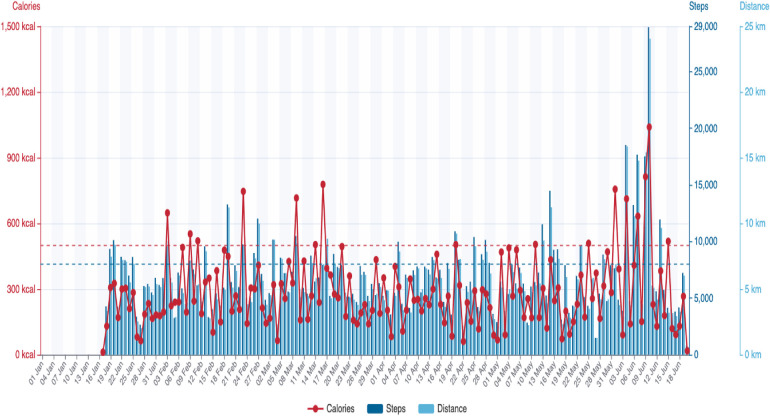
Individualised Scanwatch report.

After the baseline assessment, participants were randomly allocated into two groups: online home-based (online monitored, home-based exercise group; *n* = 15) or gym-based (in-person attendance at community-based health clubs exercise group; *n* = 15). The exercise groups followed a similar structure and exercise protocol for 6 months. A Consolidated Standards of Reporting Trials (CONSORT) flow diagram is shown in Figure 1. After the baseline assessment, each patient was provided with a watch accelerometer recorder for 7 days. All pre- and post-intervention tests were performed at the same time of day (i.e., in the morning) to minimise intra-day variability by the same assessors who were blinded to group allocation.

#### Randomisation

To ensure allocation concealment, participants were randomly allocated (stratified randomisation) by an independent statistician blinded to the study's procedures into two groups using nQuery (GraphPad Software, DBA Statistical Solutions, Boston, MA, USA) software after the baseline assessment. The stratified randomisation was based on four variables: sex (i.e., male/female), number of comorbidities (e.g., hypertension), echocardiography (i.e., LVEF), and CRF (i.e., VO_2peak_ ml/kg/min) tests. The allocated group was announced to both the principal investigator and each participant only to prevent selection bias.

## Study outcomes

### Anthropometrics

The participant's height was measured using a Hite-Rite Precision Mechanical Stadiometer. Body weight (kg), body mass index (BMI), fat mass (kg), and lean body mass (kg) were assessed at baseline and 6-month follow-up using a bio-electrical impedance analysis electrical scale (Bodystat Quadscan 4000 Touch, Bodystat Limited, Isle of Man, British Isles).

### Peak oxygen uptake test on a treadmill ergometer

During the cardiopulmonary tests, gas exchange was collected and analysed using an online breath-by-breath analysis system (Ultima™ CPX Series; Medical Graphics Corporation, Saint Paul, MN, USA). HR and ECG were continuously monitored using a Polar HR monitor (Polar FS1, Polar Electro, Kempele, Finland) and a 12-lead ECG device (CardioSoft V6.73; GE Healthcare, Chicago, IL, USA). Blood pressure (BP) was assessed during the third minute of each stage using a sphygmomanometer. Ratings of perceived exertion (RPE) were recorded during the last 10 s of each minute of the exercise test until volitional exhaustion using the Borg scale of 6–20 points. Treadmill ergometer speed and inclination and test duration were also recorded. VO_2peak_ was defined as the average oxygen consumption that was recorded from expiratory samples during the final 30 s of exercise. The respiratory exchange ratio (RER) was ≥1.10.

### Echocardiography

Echocardiography was assessed only at baseline, as this measurement was mainly used to explore the echocardiographic characteristics of our participants and ascertain balanced group randomisation. The echocardiographic assessment was performed using a Vivid S70 (GE Healthcare, Chicago, IL, USA) equipped with an M5S phased-array transducer. All examinations were stored in EchoPAC version 204 and analysed according to the guidelines of the European Association of Cardiovascular Imaging and the American Society of Echocardiography. The modified biplane Simpson method was used to assess the size and the systolic function of the left ventricle (LV). LVEF, end-diastolic volume, and end systolic volume of the LV were measured. A wall motion evaluation for segmental wall motion abnormalities was performed. The maximum volume of the left atrium was also measured. The diastolic function and LV filling pressure were assessed using early diastolic transmitral flow velocity, late diastolic transmitral flow velocity, and their ratio. Tissue Doppler imaging was used to display myocardial velocities in the lateral and septal basal regions and to calculate the average E/E’ ratio as an index of left ventricular filling pressure.

### Physical activity assessment

After the baseline and 6-month follow-up assessments, all patients were provided with a watch accelerometer recorder (Scanwatch, Withings, Issy-les-Moulineaux, France) for 7 consecutive days. Patients were requested to wear the watch accelerometer for 24 h/day except during bathing. The data were considered valid with a minimum of four weekdays and one weekend day of recordings for at least 19 h per day, which constitutes 80% of the 24 h. All data were uploaded to the online platform and stored in each patient's electronic health record. The mean daily steps, distance, and calories for each patient were estimated.

### Exercise programme

#### Design of the interventions

##### Home-based group

During the online real-time exercise session, all the haemodynamic responses and readings were continuously transferred live via the mobile application to the health instructor and cardiologist who were delivering and monitoring the session, respectively. The online session was delivered in real-time via the Zoom (https://zoom.us) web-based platform. Beyond the haemodynamic responses, participants were requested to provide the RPE levels at regular intervals during the exercise session.

#### Telemonitoring and wearable devices

The home-based group was provided with the following equipment: (1) a smart watch (Scanwatch; Withings, Issy-les-Moulineaux, France) with ECG and HR readings; (2) an oximeter assessing the saturation of oxygen; (3) an automated BP monitor; and (4) an electric body scale. Scanwatch is a validated analogue watch that allows 30 s single-lead ECG recordings and performs spot measurements of peripheral oxygen saturation (SpO_2_). HR is also measured intermittently, while physical activity (steps) is measured continuously. Scanwatch uses the following three types of sensors: (1) dry electrodes (ECG); (2) optical sensors (SpO_2_ and pulse rate); and (3) accelerometer (physical activity). Individualised reports for daily calories, steps, and distance were also recorded via Scanwatch (Figure 1). Furthermore, an online web platform was created where the cardiologists were able to log in and monitor the overall haemodynamic responses of the patients both during exercise in real-time and at rest.

##### Gym-based group

The gym-based group was requested to attend a community-based health club near their residence. The gym-based group followed an identical exercise training programme to the home-based group under the instruction and supervision of a physical education teacher experienced in cardiac rehabilitation, but without monitoring the haemodynamic parameters during the exercise sessions. The Borg RPE scale was used to assess the patients’ perceptions of their effort and exertion. The patients in both groups had to attend at least 80% of the exercise sessions offered to be included in the analysis.

#### Intensity and volume of the aerobic protocol

Based on the joint position statement of the European Association for Cardiovascular Prevention and Rehabilitation, the American Association of Cardiovascular and Pulmonary Rehabilitation, and the Canadian Association of Cardiac Rehabilitation (12), our exercise protocol was appropriately structured to fit the cardiometabolic requirements for cardiac patients. After a cardiopulmonary exercise test (CPET) on a treadmill ergometer, including VO_2peak_, ECG, BP, and HR outcomes, we devised an individualised low- to moderate-intensity aerobic protocol based on the patient's physiological responses [i.e., ventilatory thresholds (VTs) 1 and 2, ECG, BP, and HR_peak_] during the CPET.

Overall, the goal of exercise training for cardiac patients is to reach approximately 1,500 kcal/week (13), which corresponds roughly to moderate-to-vigorous intensity exercise 6 days/week. However, this level is difficult to attain by some patients, especially for those in the early post-hospitalisation phase (i.e., people with CAD after a recent MI). Early post-hospitalisation exercise training involves an attainable training dose of 20–30 min per session for 3–4 days per week (12). In addition, considering the lower fitness-lower training stimulus intensity principle, intensities even much lower than those corresponding to VT1 should be effective in cardiac patients with a markedly reduced exercise capacity (i.e., early post-hospitalisation patients). Aerobic training intensities as low as 40% VO_2peak_ (corresponding to approximately 25% VO_2_R) have demonstrated to be effective in patients with chronic HF with significantly reduced pre-training peak VO_2_ (14). Therefore, based on the above, our exercise protocol for our study participants who were considered in the early post-hospitalisation phase (i.e., recent MI) was to perform three non-consecutive sessions per week, with the duration of the aerobic protocol (i.e., aerobic music workout) at 30 min and the intensity as moderate (i.e., VT1, which marks the limit between the slight and moderate intensity of exercise).

The training principle of progression (15) is delineated as the gradual and systematic increases in the overall training dose (i.e., frequency, intensity, and/or volume) to maintain overload, thus inducing further training adaptations. In our study, the volume of aerobic exercise could not be altered due to the overall duration of each exercise session (i.e., 1 h) to assure the pragmatic conditions of our exercise programme. Therefore, to ensure the training progression of the aerobic protocol for each of our participants, the intensity was adapted based on the participant's RPE responses (for both groups) and HR recordings (for the home-based group).

#### Resistance training protocol

Resistance training (RT) combined with aerobic exercise is more beneficial than aerobic exercise alone for coronary heart disease (16). RT can effectively improve exercise capacity and a patient's QoL compared with usual care (16). Sarcopenia (i.e., loss of skeletal muscle strength, mass, and functionality) is commonly reported in CVD populations, such as in individuals with HF and CAD (17). The loss of muscle mass and strength negatively affects QoL and is an independent predictor of mortality in people with CVD (18). RT is able to induce musculoskeletal improvements, including muscle strength, power, hypertrophy, and muscle endurance.

Considering the American College of Sports Medicine's guidelines for cardiac patients (13) and a recent review of the available literature with respect to RT in cardiac rehabilitation (19), we devised our RT protocol (similar for both groups), which consisted of four main muscle group exercises (e.g., chest press, low row, lateral raising of the arms in an upright position, and chair-based bodyweight squat) that were performed using resistance bands (TheraBand, Akron, OH, USA), 1–3 sets per exercise, 90 s rest between sets, and 8–10 repetitions for each set, corresponding to an intensity of 13–15 on the RPE scale (scale of 6–20 points). The total RT duration was approximately 15 min.

Optimal progression models for hypertrophy, strength, power, and muscular endurance have not yet been delineated in people with cardiac conditions (19). In our study, to account for the training musculoskeletal adaptations to RT, we continuously monitored the RPE responses of our participants during each resistance exercise. Following consistent (>3 consecutive times) RPE responses that were below the lowest point of the target range (i.e., <13), the intensity was increasingly adjusted by altering either the participant's distance from the resistance band or the intensity of the resistance band (i.e., changing the colour of the band corresponding to a higher intensity).

#### Balance and flexibility training protocol

Both groups performed identical balance and flexibility training. More specifically, the balance training consisted of the following two balance exercises (20): (1) walking heel-to-toe (3 sets of 20 steps per set); and (2) standing on one foot (10–15 sets of 8 s for each leg). Flexibility training (21) consisted of the following stretching exercises: (1) torso stretch, (2) torso twist, (3) neck stretch, (4) quadriceps stretch, (5) hamstring stretch, (6) calf stretch, (7) shoulder stretch, and (8) chest stretch. Each stretch had a duration of 15–30 s, three to five times per side.

### Statistical analysis

Data analysis was performed using SPSS software version 23 (IBM Corp., Armonk, NY, USA) and is presented as mean ± SD. Normal distribution of the data and homogeneity of variances were tested using the Shapiro–Wilk and Levene's tests, respectively. The comparison of the anthropometric, CRF, and physical activity variables between and within the two groups (i.e., pre- and post-differences within the same group) was carried out through independent and paired-sample *t*-tests; chi-square tests were also used to identify the differences between the two groups. Pearson's correlation coefficient was performed between all variables to assess any linear correlations in our data. Statistical significance was set at *p* ≤ 0.05.

## Results

**Figure 2:**
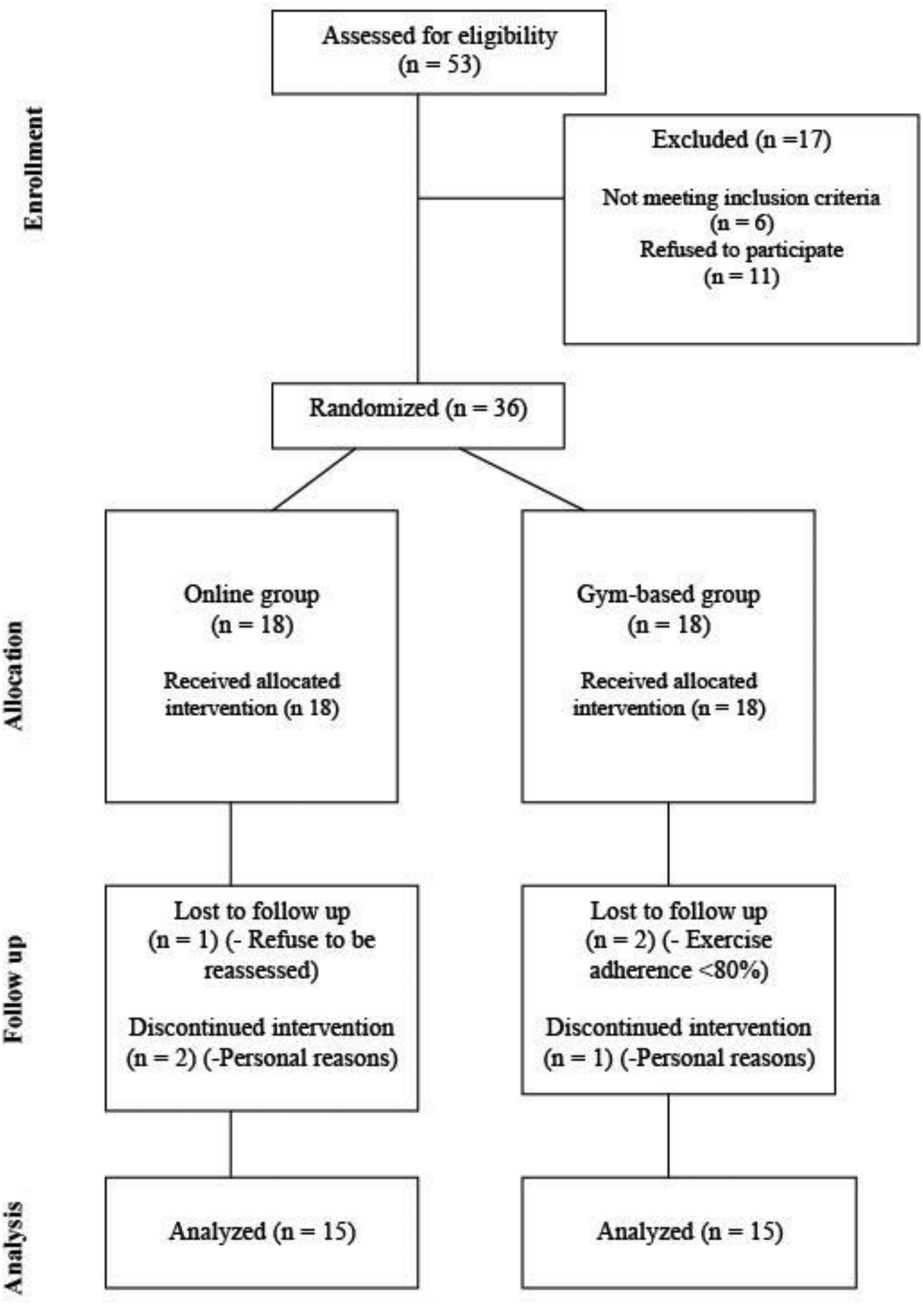
CONSORT flowchart.

A total of 53 patients with CAD after a recent MI were recruited at baseline; 6 patients did not meet the inclusion criteria and 11 declined to participate in the study. The remaining 36 patients were randomly assigned to two equally sized groups. However, three patients from each group withdrew from the study. The flowchart of participants was based on recommendations from CONSORT and is presented in Figure 2. There was no statistically significant difference between the two groups’ demographic and clinical characteristics (Table 1).

**Table 1:** Participants’ demographic and clinical characteristics.

	Online group (*n* = 15)	Gym-based group (*n* = 15)	Total (*n* = 30)	*p*-values
Age (years)	54.1 ± 8.5	53.5 ± 6.6	53.8 ± 7.5	0.85
Sex (male %)	73	73	73	
Body weight (kg)	88.5 ± 18.1	87.5 ± 12.1	88.0 ± 15.1	0.87
Height (cm)	176.5 ± 7.6	174.6 ± 7.5	175.6 ± 7.5	0.49
BMI (kg/m^2^)	28.1 ± 3.8	28.7 ± 3.0	28.4 ± 3.4	0.67
BSA	2.0 ± 0.2	2.0 ± 0.2	2.0 ± 0.2	0.66
LVEF (%)	49.5 ± 11.5	51.1 ± 9.7	50.3 ± 10.5	0.67
HR rest (bpm)	73 ± 16	70 ± 18	72 ± 14	0.56
SBP rest (mmHg)	113.57 ± 18.23	121.74 ± 15.82	117 ± 14	0.13
DBP rest (mmHg)	72.43 ± 12.04	76.21 ± 18.61	74 ± 12	0.38
Risk factors				
Hypertension, *n* (%)	5 (33.3)	4 (26.7)		0.61
Diabetes mellitus, *n* (%)	2 (13.3)	3 (20)		0.63
Dyslipidaemia, *n* (%)	5 (33.3)	7 (46.7)		0.47
Smoking, *n* (%)	8 (53.3)	5 (33.3)		0.27
Family history, *n* (%)	4 (26.7)	6 (40)		0.44
Clinical				
Time after MI (months)	3.5	3.2		
STEMI, *n* (%)	12 (80)	10 (66.7)		0.41
Anterior, *n* (%)	7 (46.7)	6 (40)		0.71
Inferior, *n* (%)	5 (33.3)	4 (26.7)		0.69
NSTEMI, *n* (%)	3 (20)	5 (33.3)		0.41
PCI, *n* (%)	14 (93.3)	13 (86.7)		0.54
CABG, *n* (%)	1 (6.7)	2 (13.3)		0.54
Medications				
Beta blockers, *n* (%)	12 (80)	13 (86.7)		0.62
Antiplatelet, *n* (%)	15 (100)	15 (100)		1.0
ACE inhibitors, *n* (%)	13 (86.7)	11 (73.3)		0.36
Statin, *n* (%)	14 (93.3)	14 (93.3)		1.00
Hypoglycaemic, *n* (%)	2 (13.3)	3 (20)		0.62

CABG, coronary artery bypass graft; DBP, diastolic blood pressure; PBF, percentage of body fat; STEMI, ST-elevation myocardial infarction; NSTEMI, non-ST elevation myocardial infarction; PCI, percutaneous coronary intervention; SBP, systolic blood pressure.

High compliance was reported concerning the watch accelerometer recorder since all participants met the valid wear time criteria (i.e., ≥80%). There were no exercise-induced adverse events during the study. Anthropometric and echocardiographic characteristics, including HR and blood pressure assessments, did not demonstrate statistically significant differences between the groups (Table 1).

The independent *t*-test revealed that at baseline, the VO_2peak_ exercise time (*p* < 0.05) for the gym-based group was of longer duration compared to the online group (Table 2). The online group showed statistically significant improvements in mean daily steps (*p* < 0.05) and mean daily distance (*p* < 0.05) at 24 weeks compared to the gym-based group (Table 2). The remaining variables did not show any statistically significant differences in between-group comparisons.

**Table 2:** Within-group comparison at baseline and 24 weeks.

	Online group (*n* = 15)		Gym-based group (*n* = 15)	
	Baseline	24 weeks	Baseline	24 weeks
VO_2peak_ (ml/kg/min)	27.2 ± 3.4	29.4 ± 3.5*	26.7 ± 2.6	29.5 ± 2.8*
Predicted VO_2peak_ (%)	85 ± 12	92 ± 13*	84 ± 8	92 ± 10*
Exercise time (min)	6.3 ± 0.7	7.7 ± 0.6*	6.9 ± 0.9	8.2 ± 1.0*
Mean daily steps	6,316 ± 1,033	9,173 ± 1,596*	6,053 ± 757	8,110 ± 1,145*
Mean daily distance (km)	5.0 ± 0.7	7.4 ± 1.3*	5.1 ± 1.1	6.4 ± 0.9*
Mean daily calories (kcal)	272 ± 122	464 ± 262*	260 ± 55	366 ± 64*
Lean muscle mass (kg)	57.7 ± 13.0	59.6 ± 12.7*	56.3 ± 6.4	58.7 ± 7.7*
Fat mass (%)	29.8 ± 6.5	28.0 ± 6.6*	29.5 ± 3.3	27.8 ± 3.3*

* *p* < 0.001; within groups comparison.

The paired-sample *t*-test showed that all the assessed variables were statistically (i.e., *p* < 0.001) improved for both groups at 24 weeks (Table 2).

Pearson's r demonstrated positive correlations between VO_2peak_ and mean daily distance (*r* = 0.375), and negative correlations between VO_2peak_ and muscle (*r* = −0.523) and fat masses (*r* = −0.460).

## Discussion

The current findings indicate that both groups significantly improved their VO_2peak_, mean daily steps, daily walked distance, daily calories, muscle mass, and fat percentage. Evidently, a 1 ml kg^−1^ min^−1^ increase in VO_2peak_ is directly associated with an approximately 10% reduction in the risk of CVD mortality (22). In our study, the Δchange (i.e., between the baseline and 6-month assessments) in VO_2peak_ was found to be 2.8 ± 1.9 and 2.2 ± 1.5 ml kg^−1^ min^−1^ for the gym-based and online groups, respectively. Therefore, the online-delivered home-based exercise programme, using digital technology to monitor the patient's physiological responses during exercise, seems to be effective in improving the CRF.

Mitochondrial biogenesis, induced by moderate-intensity aerobic exercise, is an important cellular organelle responsible for the oxidative activity of muscles (23). Considering that a minimum of 6 weeks of moderate-intensity aerobic exercise is required to improve both the size and the number of mitochondria while also increasing the capacity for resynthesis of adenosine triphosphate (24), we prescribed our exercise protocol duration (i.e., 6 resynthesis months) to secure the establishment of this physiological adaptation for our participants. Another physiological adaptation to aerobic exercise is the increase in arteriovenous oxygen difference, which is directly related to increased VO_2peak_, via greater peripheral oxygen supply, corollary to the production of catecholamines, and increased nitric oxide bioavailability (25). Cardiac function also exhibits improved performance after an intervention with aerobic exercise (26). That is, the maximal cardiac output is improved after aerobic exercise as a result of an enlargement in cardiac dimension, improved contractility, and an increase in blood volume, allowing for greater filling of the ventricles and a consequently larger stroke volume (26).

Previous evidence has demonstrated the beneficial effects of CR to induce LV remodelling in patients with LV dysfunction after MI, and the benefits were even more pronounced at the post-MI acute phase of early CR (27). Beyond early CR, though, lifelong exercise seems to have the capability to preserve LV systolic function and potentially mitigate the adverse effects of LV remodelling after MI in veteran athletes (28). In addition, patients with acute MI who underwent early CR involving two different intensities (i.e., either high-interval training or continuous moderate intensity) for 12 weeks demonstrated a significant increase in VO_2peak_ (29). Furthermore, the combination of RT and aerobic training has been demonstrated to be more effective in both strength and CRF than aerobic training alone (30). Therefore, it is evident that exercise training is able to effectively improve cardiac performance and CRF in MI patients.

Our exercise protocol was found to be effective in improving body composition by increasing lean muscle mass and reducing the percentage of fat mass in both exercise groups. In agreement with our findings, a recent systematic review indicated that RT not only could improve body composition but could also improve the haemodynamic parameters and exercise tolerance in patients with CAD (31). Thus, the RT component in our exercise programme could also promote improvements in VO_2peak_ via haemodynamic upregulation, leading to increased exercise tolerance. That is, combined RT together with aerobic training can increase maximal and submaximal exercise tolerance with reduced haemodynamic responses and a decrease in blood lactate in patients with CAD (32). Furthermore, knowing that VT is a significant exercise parameter in CAD patients, combined exercise (i.e., RT and aerobic training) is able to increase this parameter by 32.8% compared to 22.6% with aerobic training alone (32). A previous study agrees with our findings, showing that a combined exercise protocol led to a significant increase in exercise tolerance and peak oxygen consumption in patients with CAD (33). These results might point to an improvement in muscular endurance. In our study, this could potentially explain the advantageous effect of the increase in daily steps for both exercise groups. Nevertheless, the method through which these parameters improve is not fully understood. Some of the physiological responses and training adaptations that exercise could promote are changes in the vasculature, which account for the increase in muscle blood flow, improvements in lactate clearance, skeletal muscle changes, and oxidative capability after exercise.

Our findings showed an overall positive correlation between VO_2peak_ and mean daily distance, demonstrating how an improved CRF may lead to increased daily physical activity. The increased physical activity levels in CAD patients are of critical importance, with physical inactivity being adversely associated with the incidence of coronary heart disease (34). In addition, increased physical activity levels within the first year after MI were associated with reduced mortality (35). Therefore, our real-time online supervised exercise programme using wearable technology is able to increase the physical activity levels of post-MI patients with CAD.

Unlike mean daily distance, VO_2peak_ was found to be negatively correlated with muscle and fat mass. It is well known that fat mass is negatively associated with VO_2peak_ due to the physiological inability of this tissue to uptake and consume oxygen as opposed to muscle mass (36). Although we would expect a positive correlation between VO_2peak_ and muscle mass (36), our results present the opposite. Generally, VO_2peak_ declines gradually with advancing age, with a rate of approximately 10% per decade after the age of 25 years. That is, it has been reported that VO_2peak_ could decline by approximately 15% between the ages of 50 and 75 years (37). Age-related decline in VO_2peak_ results from multiple factors (e.g., decreased maximal HR and stroke volume, reductions due to heart and vascular dysfunction, and reduced peripheral oxygen extraction and maximal arterial-venous O_2_ difference) (38, 39). Another important factor in VO_2peak_ decline is age-related muscle loss (sarcopenia) by the age of 50 years, which is estimated to be approximately 10% of muscle area, and this rate demonstrates further increases in the following decades (40). Our study participants had a mean age of 54.1 and 53.5 years for the online and gym-based groups, respectively. Therefore, the negative correlation between VO_2peak_ and muscle mass could potentially be explained by the age-related loss of muscle mass.

### Limitations

A limitation of our study could be that our participants were mainly men compared to women. However, a recent systematic review and meta-analysis reported that of a total of 361 post-MI patients, 82% were men, which emphasises the prevalence of MI among men (41). Apparently, one of the risk factors for the development of this disease is sex, in addition to age (42). Oestrogen seems to be the main physiological protective factor in women. This hormone is strongly linked to cardiovascular protection (43). Apart from the sex factor, the incidence of MI appears in the 40–45 years age group. At this age, oestrogen production in women is maintained. The prevalence of MI in women is increased after menopause, when there is a significant decrease in oestrogen production (43). Another limitation of our study may be the small sample size. For this reason, our results should be interpreted with caution.

## Conclusion

Our findings indicate that a home-based online and real-time cardiac telerehabilitation programme could be as effective as a traditional gym-based cardiac rehabilitation programme (44–46). That is, the online home-based group improved CRF significantly similar to the gym-based group, thus demonstrating its effectiveness. Future studies should explore the feasibility (i.e., adherence rates, health economics, and long-term survival rates) of such a real-time online cardiac telerehabilitation programme in a large population of patients after MI.

## Data Availability

The raw data supporting the conclusions of this article will be made available by the authors, without undue reservation.
